# Expression of cytokeratin-7 and cytokeratin-19 on newborn mice induced rhesus rotavirus as biliary atresia model

**DOI:** 10.11604/pamj.2022.42.322.28408

**Published:** 2022-08-31

**Authors:** Bagus Setyoboedi, Ahmad Rofii, Anang Endaryanto, Sjamsul Arief

**Affiliations:** 1 Department of Child Health, Faculty of Medicine, Universitas Airlangga/Dr. Soetomo General Hospital, Surabaya, Indonesia

**Keywords:** Biliary atresia, cytokeratin-7, cytokeratin-19, mice model

## Abstract

**Introduction:**

biliary atresia (BA) is a progressive inflammation that causes obstruction and fibro-obliteration of the bile ducts during the perinatal period. Biliary atresia occurs in about 1 in 5000 to 8000 live births, and 50% require liver transplantation. This study aims to iinvestigate the influence of induction and duration of illness after rhesus rotavirus (RRV) exposure to changes in the expression of cytokeratin-7 (CK-7) and cytokeratin-19 (CK-19) in mice models of AB.

**Methods:**

a total of 48 Balb/c less than a day after birth was included as model of BA. The overall sample was split randomly by using the randomization table into 4 control groups and 4 treatment groups. Groups 1,2,3, and 4 composed of 24 infant mice Balb/c (each group of 6 tails) with blue color code get a placebo (buffered saline) intraperitoneallyless than a day after birth. Groups 5, 6, 7, and 8 were composed of 24 mice Balb/c (eachgroup of 6 tails) with red color code get induction RRV 1.5 x 106 Plaque forming units (PFU) as treatment groups.

**Results:**

there are influence of the RRV induced changes in the expression of CK-7 murine model of BA day 3, 7, 14 and 21 after induction compared to the control (p<0.05). There was interaction between induction effects and duration of illness after RRV exposure to CK-7 expression in murine models of BA on days 3, 7, 14 and 21 (p<0.001). There was difference in the value of CK-19 expressions progressively between trial group and control group seen from day-3 and day 21.

**Conclusion:**

induction and duration of illness after rhesus rotavirus exposure effect on the expression of cytokeratin-7 and cytokeratin-19 mice models of biliary atresia.

## Introduction

Biliary atresia (BA) results from progressive inflammation. In BA, obstruction and fibro-obliteration of the intra and extrahepatic bile ducts occur during the perinatal period [1-3]. Biliary atresia occurs in about 1 in 5000 to 8000 live births, and 50% require liver transplantation [4,5]. The incidence of cholestatic jaundice is 1 in every 2,500 infants [6].

Biliary atresia´s etiology remains uncertain. There are several factors thought to play a role in the pathogenesis of biliary atresia BA include infection, fetal circulatory disorders, abnormal morphogenesis, exposure to toxins, and immunological disorders [4,7,8]. Perinatal hepatobiliary viral infection will cause premature apoptosis or necrosis cholangiocyte if it lasts a chronic inflammatory process will lead to the total destruction of the bile ducts [7,9]. In BA, there is cell damage caused by an immunological response. In addition, there is a change in the characteristics of bile duct cells to those of mesenchymal cells.

Bile duct epithelial cells lose cell polarity, cell communication, loss of normal structure of epithelial cells accompanied by the accumulation of extracellular matrix (MES) [10-12]. This process is known as epithelial mesenchymal transition (EMT) [13-16]. In the process of EMT is characterized by decreased expression of calcium-dependent adhesion epithelial (E-cadherin), cytokeratin-7 (CK-7), cytokeratin-19 (CK - 19), integrins and increased expression of calcium-dependent adhesion neural (N-cadherin), vimetin, fibronectin, α - smooth muscle actin (α-SMA), fibroblast specific protein-1 (FSP-1) [17-19]. Studies in cell culture defined decreasing of epithelial cells markers such as cytokeratins and increasing of mesenchymal cells markers [20-22]. Liu (2013) found decreased expression of E cadherin and increased expression of N-cadherin (cadherin switch) day 6 after induction of TGF-β in cell culture bile duct and De Vries (2011) found an increase in CK-7 and CK-19 day 7 and 14, but Taura (2010) in vitro did not get get an increase in markers of mesenchymal cells [17,23,24]. The purpose of this study is to investigate the influence of induction and duration of illness after rhesus rotavirus (RRV) exposure to changes in the expression of CK-7 and CK-19 in mice models of AB.

## Methods

This research is purely experimental research using factorial design to investigate the influence of induction and duration of illness after rhesus rotavirus (RRV) exposure to changes in the expression of CK-7 and CK-19 in mice models of AB.

**Subject:** a total of 48 Balb/c less than a day after birth was included as model of BA. The overall sample was split randomly by using the randomization table into 4 control groups and 4 treatment groups. Groups 1,2,3, and 4 composed of 24 infant mice Balb/c (each group of 6 tails) with blue color code get a placebo (buffered saline) intraperitoneallyless than a day after birth. Groups 5, 6, 7, and 8 were composed of 24 mice Balb/c (each group of 6 tails) with red color code get induction RRV 1.5 x 10^6^ PFU as treatment groups. Inclusion criteria of Balb/c mice newborn from healthy carries, aged less than a day after birth startup research, and no any physical deformities. We excluded the baby mice born premature, and we drop out the mice with damage organs or tissues at the time of sampling for examination or the baby mice dead before age termination as specified. The control groups (O1) given 50 μL buffered phosphate saline intraperitoneally less than a day after birth, then termination performed on the days 3, 7, 14 and 21, then checked the variables (P1-P4). Less than a day after birth, treatment groups (O2) were induced 50 μL of phosphate buffered saline containing 1.5 x 10^6^ PFU RRV intraperitoneally. Furthermore randomly selected for termination performed on days 3, 7, 14, and 21, then examined the variables (P1-P4).

**Setting:** this research was conducted at the Laboratory of Biomolecular and Biomedical Laboratories, Faculty of Medicine, University of Brawijaya, Malang. The data collection of this research was conducted in January-February 2014.

**Data analysis:** analysis of the average for numerical data using mean and standard deviations (SD) of each variable with the independent sample t-test to the data with univariate normal distribution. For the results of the study were not normally distributed, the analysis performed by Mann Whitney test (for data with 2 groups) or Kruskal-Wallis (for data with >2 groups) for each group by using the median, respectively.

**Ethical clearance:** this study is part of a larger research entitled “benefits and the effect of corticosteroids on the difference between the expression of CD56, CD68, TLR, NFkB, CD4, CD8, ANCA, fibrosis and bile duct obstruction in animal model of biliary atresia (study changes in innate immune response, innate to adaptive non-self, adaptive non-self and adaptive self)” who has received information ethics from the Health Research Ethics Committee of the Faculty of Medicine, University of Brawijaya.

## Results

**Figure 1:**
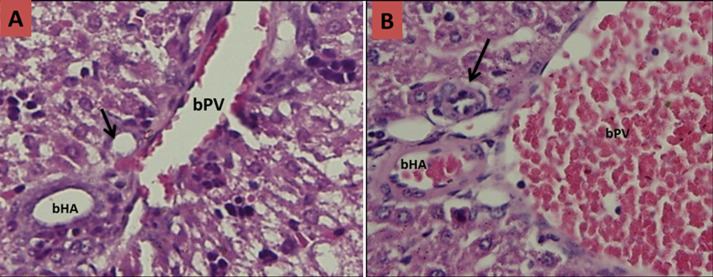
histological slices of liver and biliary tract tissues of infant mice after termination day 3; A) control groups: no visible presence of inflammatory cells in the lumen of the bile duct with no narrowing (black arrow); B) treatment groups: visible infiltration of inflammatory cells accompanied by swelling of the lumen of the bile duct that causes ranging narrowed lumen (black arrow) (bPV: branch of portal vein, bHA: branch of hepatic artery)

**Figure 2:**
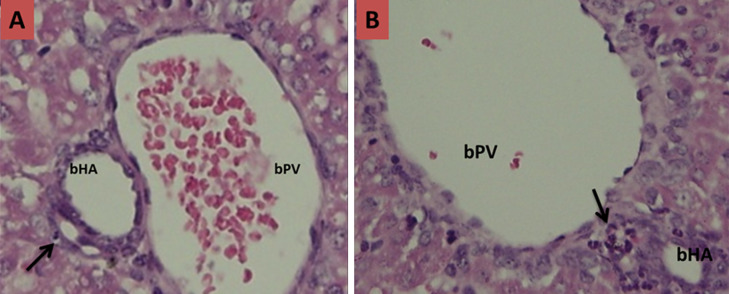
histological slices of liver and biliary tract tissues of infant mice after termination day 7; A) control groups: no visible presence of inflammatory cells in the lumen of the bile duct with no narrowing (black arrow); B) treatment groups: visible infiltration of inflammatory cells that multiply the lumen of the bile duct causing swelling that result in increased narrow lumen (black arrow) compared to day 3 (bPV: branch of portal vein, bHA: branch of hepatic artery)

**Figure 3:**
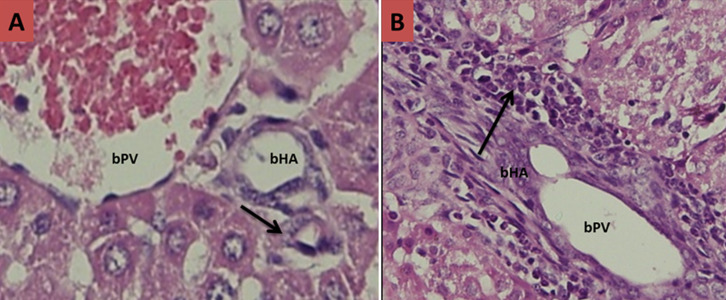
histological slices of liver and biliary tract tissues of mice after termination day 14; A) ontrol groups: no visible presence of inflammatory cells in the lumen of the bile duct with no narrowing (black arrow); B) treatment groups: visible infiltration a lot of inflammatory cells that cause swelling and the lumen of the bile ducts become clogged lumen (black arrow) (bPV: branch of portal vein, bHA: branch of hepatic artery)

**Figure 4:**
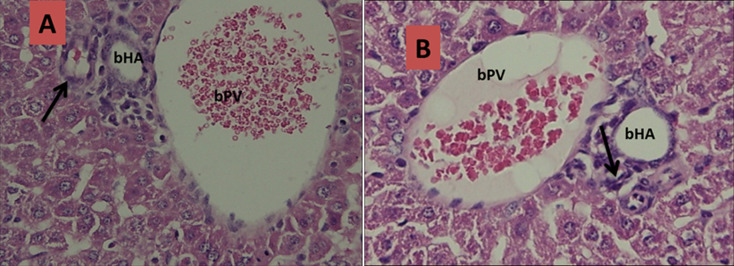
histological slices of liver and biliary tract tissues of mice after termination day 21; A) control groups: no visible presence of inflammatory cells in the lumen of the bile duct with no narrowing (black arrow); B) treatment groups: visible lumen of the bile duct has undergone atresia (black arrows) (bPV: branch of portal vein, bHA: branch of hepatic artery)

Basic characteristic of neonatal mice in each trial groups can be seen in Table 1. The baseline characteristics of weight initially homogeneous sample because the sample preparation in accordance with the inclusion criteria. However, in general there are differences in the mean body weight of mice when performed late termination (not included in the table). The increase in body weight of mice in the trial groups comparatively lower than the control groups for each day of termination. In this study, histopathological examination was also carried out to prove the occurrence of biliary atresia in mice induced RRV. Here is a comparison picture of the biliary tract histopathology qualitatively compared between the treatment groups to control groups (Figure 1, Figure 2, Figure 3 and Figure 4).

**Table 1:** sample characteristics

Day	Control groups				Trial groups			
	3	7	14	21	3	7	14	21
Number of groups	6	6	6	6	6	6	6	6
Mean early BW (g)	1.84	1.82	1.80	1.81	1.85	1.83	1.80	1.82
Drop out	2	0	0	0	2	2	1	2
Total samples	4	6	6	6	4	4	5	4

BW: body weight

Induction RRV influence on expression of cytokeratin-7 and cytokeratin-19: from the expression of CK-7 and CK-19 biliary tract in the control groups of healthy mice that expresses all the variables studied, both CK-7 and CK-19. This suggests that the constitutive variables studied have been produced prior to the induction of RRV. To get an overview of the effect of RRV induced changes in the expression of CK-7 and CK-19, displayed the flow cytometry results in tabular form, all the results are arranged in the form of graphs that reflect the relative position of the two groups in each variable.

Induction RRV influence on expression of cytokeratin-7: in this study, a trial groups with a median value of expression of CK-7 quantitatively lower in the trial groups than the control groups with general results p<0.05, which shows the influence of the RRV induced changes in the expression of CK-7 murine model of BA day 3, 7, 14 and 21 after induction compared to the control (Table 2). Also obtained results that are interaction between induction effects and duration of illness after RRV exposure to CK-7 expression in murine models of BA on days 3, 7, 14 and 21 with p<0.001 (Table 2).

**Table 2:** changes in the expression of cytokeratin-7 in the RRV induction groups and control groups

Variable	Day	Control groups	Trial groups	p* between groups per variables
		Median (interquartil)	Median (interquartil)	
CK - 7	3	4.5 (0.06) %	4.2 (0.05) %	0.00*
	7	6.9 (0.15) %	3.9 (0.10) %	0.00*
	14	20.8% (0.08) %	10.2 (3.22) %	0.00*
	21	25.2 (0.63)%	7.7 (0.71) %	0.00*
	p*	0.000**	0.02**	

* significant differences by Mann Whitney test at α=0.05; ** significant differences by Kruskal Wallis test at α=0.05

**Figure 5:**
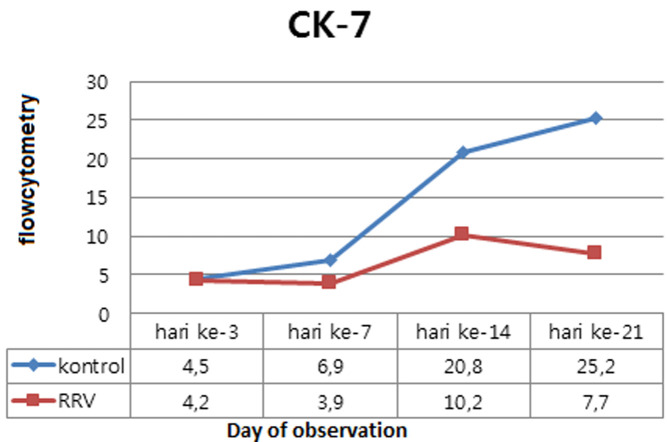
effect of induction of RRV and duration of illness after RRV exposure to changes in the expression of cytokeratin - 7 (median) compared to the control groups

The following chart changes in expression of CK-7 (median) from time to time in the control groups compare with trial groups (Figure 5). From the above chart, expression of CK-7 decreased in trial group since day 3 compared to control group. There was difference in the value of CK-7 expressions progressively between trial group and control group obtained from day-7 and the large difference in expression values obtained on day 21. The CK-7 expressions are lower in the trial group than control group.

Induction RRV influence on expression of cytokeratin-19: in this study, a trial groups with a median value of expression of CK-19 quantitatively lower in the trial groups than the control groups with general results p<0.05, which shows the influence of the RRV induced changes in the expression of CK-19 murine model of BA day 3, 7, 14 and 21 after induction compared to the control (Table 3). Also obtained results that are interaction between induction effects and duration of illness after RRV exposure to CK-19 expressions in murine models of BA on days 3, 7, 14 and 21 with p<0.001 (Table 3).

**Table 3:** changes in the expression of cytokeratin-19 in the RRV induction groups and control groups

Variable	Day	Control groups	Trial groups	p* between groups per variables
		Median (interquartil)	Median (interquartil)	
CK - 19	3	6.4 (0.64) %	5.3 (0.06) %	0.007***
	7	18.2 (0.14) %	4.3 (0.09) %	0.000*
	14	19.4 (1.02) %	8.6 (1.59) %	0.000*
	21	23.3 (0.06) %	15.7 (0.71)%	0.000*
	p	<0.000**	0.002**	

* significant differences by Mann Whitney test at α<0.05; ** significant differences by Kruskal Wallis test at α<0.05; *** significant differences byT-Test at p<0.05

**Figure 6:**
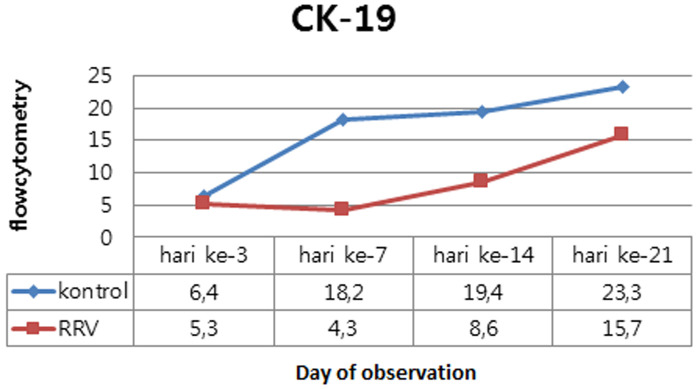
effect of induction of RRV and duration of illness after RRV exposure to changes in the expression of cytokeratin - 19 (median) compared to the control groups

The following chart changes in expression of CK-19 (median) from time to time in the control groups compare with trial groups (Figure 6). From the above chart, expression of CK-19 decreased in trial group since day 3 compared to control group. There is a progressive difference in the expression of CK-19 between the trial group and the control group obtained from day 3 and day 21. The CK-19 expressions are higher in trial group than control group. RRV induction dose of 1.5 x 10^6^ PFU intraperitoneally in infants Balb/c mice less than 1 day after birth would affect changes in cellular adaptive immune response in biliary tract tissue. In this study, the cellular adaptive immune response that occurs is reflected in the presence of expression of CK-7 and CK-19.

The hypothesis that pro-inflammatory cytokines important for the pathogenesis of BA has been tested on mice models induced RRV. Bile duct damage initiated by viral infection which is followed by the release of antigens “self” that has changed and activate autoreactive T-cells and specific bile ducts, which causes chronic fibrosclerosing injury to the bile duct [4].

## Discussion

In this study, there were differences in mean final body weight (at termination) of mice in the control groups and trial groups. Newborn mice RRV-induced increased weight gain was relatively lower than control mice because of illness. Similarly, the mortality rate of mice in the trial groups occurs more frequently, especially before day 7 and between day 14 and 21. Mortality is caused by the baby mice looking sick and eaten by its parent in accordance with their nature. Baby mice that sick is most likely due to the induction of RRV as evidenced by the death of the trial groups more than the controls. These results are similar with Allen and Bessho, who obtained more than 80% of mice that have undergone RRV-induced biliary atresia on the 14^th^ day of termination [25,26]. Petersen in his research got the lethality number reaches 100% in neonatal mice intervention after day 21 post-induction [27].

In this study, histopathological examination was done to prove the occurrence of inflammation and biliary tract obstruction as a result of an immunological response after induction of RRV. From the histopathology, inflammatory processes occurred from day 3 after induction of RRV were intensified on day 7. Obstruction process of biliary tract visible on day 14, and total obstruction seen at day 21. These results do not vary with Carvalho in which the inflammatory process in the biliary tract occurs on days 3 and 7 after induction of RRV, but he gets the total obstruction on day 14 [28]. Rhesus rotavirus infection in the biliary tract causing immunological dysregulation that results in inflammation of the duct epithelium and lumen of the duct becomes swollen. This inflammatory process is chronic and progressive because the autoimmune process that ended in the total obstruction of biliary tract and biliary tract cannot function properly. Rhesus rotavirus induction dose of 1.5 x 10^6^ PFU intraperitoneally less than 1 day after birth in infants Balb/c mice would affect changes in cellular adaptive immune response in biliary tract tissue. In this study, the cellular adaptive immune response that occurs is reflected in the presence of expression of CK-7 and CK-19. The hypothesis that pro-inflammatory cytokines important for the pathogenesis of BA has been tested on mice models induced RRV. Bile duct damage initiated by viral infection which is characterized decrease CK-7 and CK-19.

**Cytokeratin-7 expressions:** this study is an experimental study with Balb/c mice which proves that RRV induction dose of 1.5 x 10^6^ PFU intraperitoneally at the age of less than 1 day after birth provides significant effect on the decrease in the expression of CK-7 when compared with controls and the decrease is going according to the time sequence begins after day 3 post-induction RRV.

In this study, decreased expression of CK-7 in experimental group compared to control group (p <0.001). The results of the study Harada (2009) with the dsRNA analog administration on bile duct epithelial cell cultures also have reduced expression of CK-7 as well Valdes (2002), Wistar rats were induced by TGF-β that. There are differences in the expression of CK-7 were significantly (Δ = 0.30, p = 0.00) between groups of families and groups try although the difference is not too big on day 3 [21,29]. On day 7 after induction of RRV also found decreased expression of CK-7 and expression differences greater than the control group (Δ = 3.06, p = 0.00). These results are in accordance with the histopathology that showed an increase in inflammatory cells and bile duct lumen is narrower. Erickson (2008) also got mild to moderate obstruction and inflammation around the bile ducts in the preparation of histopathological on day 7 [30].

Choi (2009) found the lowest value CK-7 expressions at day 7 after administration of CCl4 in intraperitoneal in mice. Increased expression of this cytokeratins according to De Vries (2011) caused the entire cell stress induced RRV and some cells undergo phenotypic changes, especially for the formation of connective tissue, whereas the expression of CK-7 and CK-19 were decreased [24,31]. On day 14 the expression values obtained difference between experimental and control group greater (Δ = 10.6, p = 0.00) and on day 21 the greatest decrease obtained in this study (Δ = 17.5, p = 0.00). This phenomenon indicates that the longer the exposure after induction of RRV, epithelial cell damage or apoptosis, fibrosis and EMT that occurs more widely. In the histopathological results obtained biliary tract total obstruction on 21^st^ day. These results corresponded to Feng´s (2005) gain on day 21 of total obstruction extrahepatic bile duct after induction RRV [32].

Cytokeratin-7 expressions from day 3 until day 21 had an upward trend despite the lower expression values when compared with control group. This could be because the baby bulb/c is still in a growth phase so that the process of epithelialization occurs for the growth of tissues and organs as well as for cell regeneration. In bulb/c infants, the given induction RRV also in the maturity phase of growth for organ function and regeneration of cells, although the value is lower than the control group therefore occur RRV-induced inflammatory process so that the process of epithelialization of epithelial cells disrupted by inflammation, apoptosis and changes into mesenchymal cells. The difference in the value of CK-7 expressions on experimental group apoptosis and cell survival changes the phenotype of epithelial cells into mesenchymal cells accompanied by activation of fibroblasts as a result of inflammatory responses by induction of RRV.

**Cytokeratin-19 expression:** in this study, gain reduction the expression of CK-19 in experimental group (p <0.001) and the longer the exposure time, the virus was also found reduced expression of CK-19 greater than the experimental group than the control group. Harada (2009) found that after administration of the dsRNA analog bile duct epithelial cell culture, a decline in CK-19 and other markers of epithelial cells and an increase in markers - markers of mesenchymal cells, such as FSP-1 and vimentin [21]. On the third day there is a decrease in the expression of CK-19 significant (Δ = 1.1; p = 0.007) between the groups. On day 7 obtained the lowest decline and greatest expression differences CK-19 among experimental group compared to control group (Δ = 13.9; p = 0.000). Yabushita (2001) found a decrease in the expression of CK-19 since day 3 in rats induced RRV. De Vries (2011) found that difference expression of CK-19 were increased compared to control on day 7, results this is supported by the increased expression of mesenchymal cell markers such as α-SMA [24,33].

Increased expression of CK-19 at experimental group happened from day 14 (Δ = 10.8; p = 0.000) and continued on day 21 although it remained below the pattern control group (Δ = 7.6; p = 0.000). Histopathological results at day 14 showed that the distribution of inflammatory cells reached a maximum and on day 21 had occurred total obstruction biliary tract. This research is in accordance with Paku (2005) which says that although there has been a total blockage on histopathologic examination, however CK-19 can still be detected on flow cytometry examination [14,17,21].

In the EMT process biliary atresia besides a decline in the expression of CK-7 and CK-19, obtained also a decrease other markers of epithelial cells (E-cadherin) and an increase in markers of mesenchymal cells such as N-cadherin vimentin, FSP-1, α-SMA, fibronectin [10,13,20,21,29]. In biliary atresia EMT process, in addition to decrease expression of CK-19 and other epithelial cell markers, also found an increasing in the expression of mesenchymal cells (vimentin, FSP-1, α-SMA, collagen type 1, fibronectin). And in normal mammalian life, mesenchymal cells will always be formed to establish and maintain the integrity of connective tissue organs and tissues [34].

## Conclusion

The results of this study provide additional evidence of the truth of the hypothesis that the induction of RRV resulted in changes the expression of cytokeratin-7 and cytokeratin-19 in the pathogenesis of BA, thus opening discourse to do further studies for new strategies in the management of medically BA. Progressive decrease expression of cytokeratin-7 and cytokeratin-19 beginning on day 7 with a peak at day 21 shows that the possibility of a good time for medical intervention performed around day 7 and before day 14, because after day 14, the occurrence of BA already irreversible.

### What is known about this topic

- Biliary atresia (BA) is a progressive inflammatory obstruction in bile ducts during the perinatal period;
- Etiology of biliary atresia is still unclear;
- In biliary atresia, there are decreased expression of calcium-dependent adhesion epithelial (E-cadherin), cytokeratin-7 (CK-7), and cytokeratin-19 (CK-19).

### What this study adds

- There is influence of the RRV induced changes in the expression of CK-7 murine model of BA day 3, 7, 14 and 21 after induction compared to the control;
- There are interaction between induction effects and duration of illness after RRV exposure to CK-7 expressions in murine models of BA on days 3, 7, 14 and 21;
- Induction and duration of illness after rhesus rotavirus exposure effect on the expression of cytokeratin-7 and cytokeratin-19 mice models of biliary atresia.
